# Elderly Patient Care: A New Reality of the SARS-CoV-2 Pandemic

**DOI:** 10.14336/AD.2021.0222

**Published:** 2021-10-01

**Authors:** Beata Jankowska-Polańska, Kathie Sarzyńska, Aleksandra Pytel, Damian Izbiański, Dagmara Gaweł-Dąbrowska, Brygida Knysz

**Affiliations:** ^1^Division of Nervous System Diseases, Department of Clinical Nursing, Faculty of Health Science, Wroclaw Medical University, Wrocław, Poland.; ^2^Safety and Crisis Management Department, Legnica, Poland.; ^3^Department of Social Medicine, Faculty of Medicine, Wroclaw Medical University, Wroclaw, Poland.; ^4^Department of Infectious Diseases, Hepatology and Acquired Immune Deficiencies, Wroclaw Medical University, Wrocław, Poland.

**Keywords:** COVID-19, SARS-CoV-2, prevention

## Abstract

Poland is among the European countries currently facing the second wave of the SARS-CoV-2 pandemic. A lot of studies confirm the mortality rate of COVID-19 increases with age. Considering the particularly adverse outcomes of a SARS-CoV-2 infection, preventing infections should be the priority for public health professionals. One method for preventing SARS-CoV-2 infections among eldery people may involve implementing procedures for limiting the spread of the pathogen, and providing education to medical staff, so as to bridge any gaps in knowledge on virus spread and post-infection or post-exposure management. Three residential medical centers in Poland were selected for evaluation of existing medical procedures to identify any errors in the current procedures applied for the prevention of SARS-CoV-2 spread in the facility. The project involved 4 steps: (1) Audit of the existing medical procedures; (2) Knowledge evaluation for the staff (n=185) in the senior- and disabled care facilities, administration of a knowledge test developed by the authors to assess knowledge on SARS-CoV-2 prevention; (3) Training for medical staff; (4) Updates and implementation of procedures. The knowledge test conducted revealed a lack of knowledge of medical personnel about SARS-CoV-2. The deficiencies of the surveyed group varied depending on the place of employment. Almost half of the surveyed medical centers (center No. 1 (52%) vs. center No. 2 (44%) vs. center No. 3(59%)) believed that elderly people usually do not show symptoms of SARS-CoV-2 infection. In the facility No. 1, 70% of respondents did know that SARS-CoV-2 can be transmitted via the alimentary route compared to center No. 2 and No. 3 where knowledge about it showed only 28,9% and 24,8% responders, respectively. Also, in facility No. 1, the least among the studied group (67%) knew that people with comorbidities were at risk of covid-19 compared to respondents from facility No. 2 and 3, where, respectively, 100% and 93% had such knowledge. Only 33.3% of the staff of facility No. 1 knew how to deal with a patient who will present symptoms such as temp >38 degrees with coughing or dyspnea while in institution No. 2 and 3, this knowledge was slightly higher at 44% and 60% respectively. The audit of the existing hygiene procedures used to limit the risk of SARS-CoV-2 spread demonstrated a number of shortcomings. Employees at the residential medical care centers included in the study had gaps in knowledge on the spread of SARS-CoV-2. The training sessions implemented at the next stage improved knowledge on SARS-CoV-2 infections. Additionally, decisions were made to modify certain procedures and introduce new ones to better prevent the spread of SARS-CoV-2.

The first human coronavirus strains were identified in the 1960s [1]. In 1962, the HCoV-229E strain B814 was isolated from a child with symptoms of the common cold [2]. Other, much more dangerous, human coronavirus species were isolated in 2002 (SARS-CoV) and 2012 (MERS-CoV) [3]. Both these beta-coronavirus outbreaks caused severe acute respiratory distress syndromes, with a mortality rate of 9.6% for SARS-CoV [4] and 36% for MERS-CoV [5]. In 2019, a new coronavirus, SARS-CoV-2, was identified, causing a disease named COVID-19. SARS-CoV-2 bears much philogenetic and clinical resemblance to SARS-CoV but is characterized by greater transmissibility and lower mortality [4]. Since the virus is highly infectious, transmissible even during the asymptomatic phase, and has a relatively low virulence, it spread rapidly across geographical regions, resulting in a pandemic [6]. The extensive spread of the virus currently poses a major challenge for healthcare systems and governments around the world [7-9]. Less than a year after the virus first appeared, i.e., on November 10, 2020, WHO reported there had been 50 676 072 confirmed cases of COVID-19, including 1 261 075 deaths (https://iris.wpro.who.int/bitstream/handle/10665.1/14519/covid19-20201111.pdf, Accessed 5 Dec 2020). Regional data indicate the largest number of cases in the Americas (27,754,113) and in Europe (19,535,185). In Poland, between January 3 and December 5, 2020, there were 1,028,610 confirmed cases of COVID-19, with 18,828 deaths (https://covid19.who.int/, Accessed 5 Dec 2020).

Based on the data collected, researchers have identified several groups of symptoms associated with COVID-19. The most commonly observed ones include flu-like symptoms (with fever, chills, fatigue, and cough), cold-like symptoms (with rhinitis, sneezing, sore throat, and nasal congestion), joint and muscle pain, conjunctivitis, pulmonary symptoms (pneumonia, dyspnea), gastrointestinal symptoms (including diarrhea, nausea, and headaches), and loss of taste and smell [10-11]. Some patients may also experience delirium. Therefore, acute states of confusion accompanied by fever should be considered an early symptom of the disease, especially in elderly patients [12]. A rapid and well-coordinated innate immune response is the first line of defense against viral infection, though in some cases, immune regulatory mechanisms may falter, culminating in the breakdown of self-tolerance, resulting in immune-mediated attack directed against both viral and self-antigens [13]. Researchers have distinguished several clinical phenotypes of the disease caused by SARS-CoV-2, based on symptom severity and patient condition, with degree of hypoxemia used as the measure of severity [14].

There is no clear evidence of increased infection risk among elderly patients, but old age is reported as an independent risk factor both for death from SARS-CoV-2 and for a severe course of infection. Patients aged over 60 years and having serious preexisting conditions have been demonstrated to be at a higher risk of acute respiratory distress syndrome and death [15]. In a report by Chinese researchers, a higher infection rate among the elderly was not clear, but their analyses did demonstrate a higher mortality in this age group. According to a report from a Chinese center for disease control, the number of COVID-19 cases (confirmed by tests for SARS-CoV-2 and classified based on symptoms) in the country was 72,314 by February 11, 2020. Based on the collected data, the mortality rate in this group was 2.3%, but increased to 8% in the 70-79 years age bracket and reached 14.8% in patients aged 80 and above [16].

Italian data confirm the association between older patient populations and higher mortality. During the first wave of the pandemic in Italy, the overall mortality rate was 7.2%, and patients aged 70 and above accounted for as many as 37.6% of all cases. Risk factors for severe COVID-19 include concurrent chronic diseases, which are often typical of older age. Older patients are also more likely to have multiple such diseases. Among the chronic diseases found to occur two or three times more often in COVID-19 patients, the most common ones include diabetes mellitus, high blood pressure, and ischemic heart disease. Research findings indicate that the risk of death is significantly higher in patients with these concurrent diseases than in those who have COVID-19 without comorbidities. Cardiovascular disease increases the risk of severe COVID-19 forms and death [17].

The latest epidemiological data from Poland, dated December 6, 2020, confirm the higher mortality from SARS-CoV-2 infection in older patient groups. The rates were: 7.1% for patients aged 60-70, twice as high — 15.2% — in those aged 70-80, and the highest in those aged 80 or above — 18.7% [www.pteilchz.org.pl/wp-content/uploads/2020/12/%C5%9Amiertelno%C5%9B%C4%87-w-COVID-19-SARSTer-6-12-2020.pdf, Accessed 5 Dec 2020]. The higher risk of death in elderly patients does not only result from comorbidities. Other contributors may include immunosenescence and malnutrition, found to contribute synergistically to the greater susceptibility of the elderly to SARS-CoV-2 and the worse outcomes [18]. Therefore, especially in the elderly, rapid identification and limitation of SARS-CoV-2 spread is essential. Though the virus primarily causes respiratory infections, it has adverse effects on many other organs, causing acute cardiomyopathies, chronic cardiovascular dysfunction, and multi-organ failure with cytokine storm [6,7].

Current evidence suggests that the virus is mainly transmitted by respiratory droplets. Transmission via aerosol is possible under certain conditions, especially in confined, crowded, and poorly ventilated spaces, where the infected person spends a great deal of time with others. Infection can also occur during patient care if procedures causing the formation of aerosol are performed. The virus can be spread when infected individuals sneeze, cough, or touch surfaces or objects such as tabletops, railings, or doorknobs. Other people can contract the virus by touching these contaminated surfaces, and then touching their eyes, nose, or mouth without having washed their hands [19].

Based on the transmission mode of SARS-CoV-2 and the risks associated with SARS-CoV-2 infection in the elderly and those with comorbidities, a number of preventive measures have been recommended. Reducing the SARS-CoV-2 infection risk in groups particularly susceptible to its adverse outcomes should become the priority for public health professionals. Particular attention should be paid to all senior living facilities (www.cdc.gov/coronavirus/2019-ncov/healthcare-facilities/prevent-spread-in-long-term-care-facilities.html, Accessed 5 Dec 2020): in Poland, these facilities are classified as residential medical care centers, residential rehabilitation care centers, or nursing homes.

The Polish Deputy Health Minister, Józefa Szczurek-Żelazko, said in a statement that between the start of the pandemic and September 15, 965 patients of residential medical or rehabilitation care centers were infected, accounting for approximately 2.4% of patients using these services annually (out of a total of approx. 40,700 patients annually)(http://orka.sejm.gov.pl/zapisy9.nsf/0/D4CDBDFBC8AE52ADC125860300234B36/%24File/0062309.pdf, Accessed 5 Dec 2020). In hospices, 66 patients were infected, or approx. 0.2% of patients treated in hospices annually. Among staff, the number of infections was 535 in residential care centers and 46 in hospices [20]. Due to the dynamic development of the COVID-19 situation in the Polish population, preventive measures have been taken to reduce the spread of SARS-CoV-2 in selected long-term care facilities.

## MATERIAL AND METHODS

The study was conducted in 3 public Residential care and treatment centers located in the same region (Legnica) with permanent residents over the age of 65 years. Medical employees (nurses, medical carers, physiotherapists and doctors) of these centers were asked by managers to fill in a paper of a self-administered questionnaire, prepared by the authors, concerning general knowledge about covid-19 and ways of spreading the SARS-CoV-2 virus. All employees signed a consent to participate in the study. 5% of the questionnaires were rejected due to significant shortcomings in the answers provided. The study was approved by the Bioethics Committee of the Wroclaw Medical University No. 52/2021.

**Figure 1.**
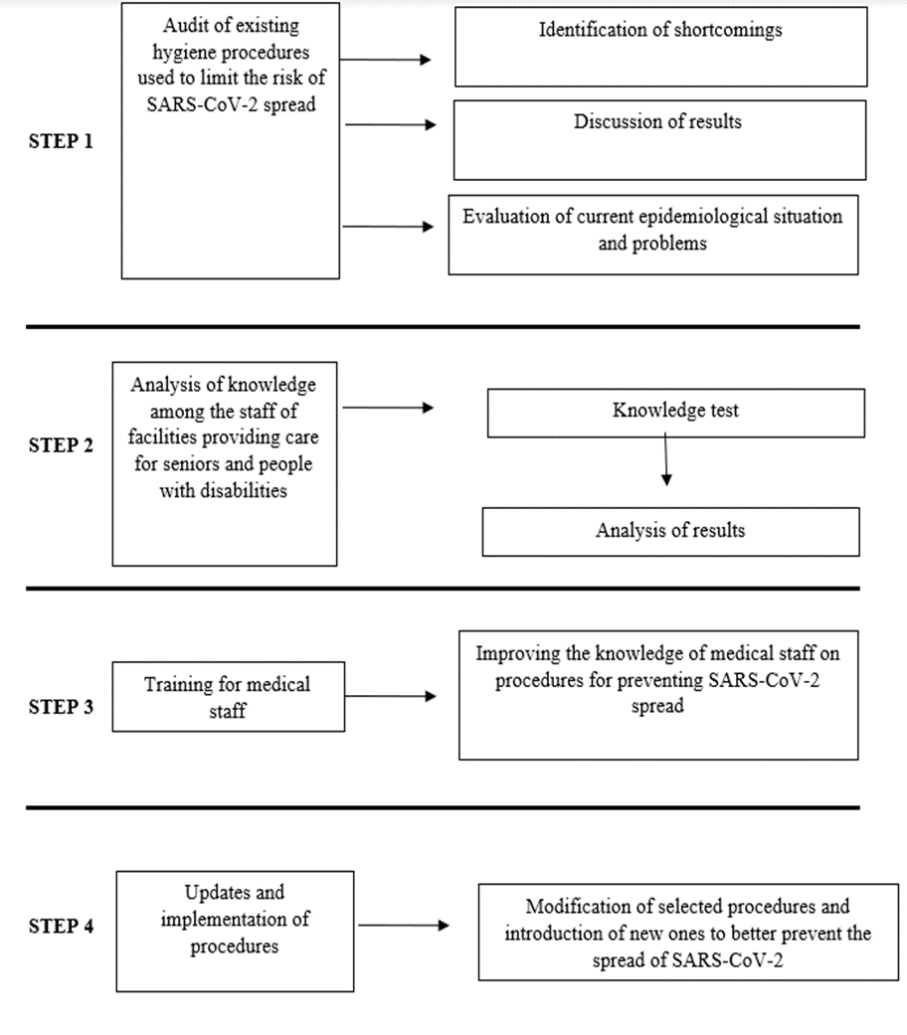
Study flow diagram.

Statistical analysis was performed using the Statistica 13 program (TIBCO, Inc., USA). For measurable variables, arithmetic means and standard deviations were calculated. The frequency of occurrence (percentage) was calculated for qualitative variables. All investigated quantitative variables were checked with the Shapiro-Wilk test to determine the type of distribution. The comparison of qualitative variables between the groups was made using the chi-square test (χ2). The comparisons of the results depending on the place (place 1, 2, 3) were performed using the one-way analysis of variance (ANOVA) test and the post-hoc test (Tukey’s test). The level of α = 0.05 was assumed for all comparisons. Measures taken (Fig. 1):

1)Audit of existing hygiene procedures used to limit the risk of SARS-CoV-2 spread.

Discussion of strategic objectives and action plans with the management- The operation of residential medical care centers and nursing homes is regulated by the Polish Social Welfare Act and supervised by the Ministry of Family and Social Policy. Though these facilities provide care, nursing, medical, and rehabilitation services, qualified medical personnel only form part of the staff they employ. The facilities are not subject to health and epidemiological surveillance, which means that their plan for preventing infection risk may be developed and supervised by individuals without sufficient medical expertise. In contemporary medicine, control of infectious diseases and hospital-acquired infections is considered one of the most important challenges. Infections can be successfully limited thanks to effective infection control methods. In a hospital, there is an organizational unit that monitors cases of infection, called the Hospital Infection Control Team, led by a nurse epidemiologist who manages a program for hospital-acquired infection prevention and control. One of the areas of surveillance deals with hygiene maintenance in medical facilities. Among the institutions analyzed here, only one employed a nurse epidemiologist, and did so only part-time. In the other two, epidemiological surveillance was handled by individuals with no background in epidemiology. In day-to-day infection surveillance in healthcare settings, the leading role is played by prevention, which largely depends on the effectiveness of hygienic procedures in place. Periodic evaluations of cleaning effectiveness are performed by sanitary inspectors. However, routine supervision of cleaning and disinfection must be performed in the healthcare facility. Ongoing supervision is carried out by head nurses or heads of organizational units, in accordance with the hygiene plan and procedures of each facility. The cleanliness of surfaces in a hospital is typically evaluated at least once a month. If a patient or a member of the medical staff comes into contact with contaminated surfaces, equipment, or tools, there is a risk of microbial transmission and epidemic outbreaks. The hospital environment is a reservoir of pathogens, which is why all the hygiene procedures must be carried out correctly. The hands of medical personnel are the primary vector of healthcare-associated infections. Procedures for hygienic hand washing and disinfection and for surgical hand scrubbing have been in place in Poland for many years. The WHO guidelines on hand hygiene, applied in Poland, define situations that require hand hygiene, along with specific procedures for hand disinfection. The methods currently used for combating infections involve creating a medical environment unsuitable for the survival and replication of pathogenic microorganisms, preventing the transmission (dislocation) of pathogenic micro-organisms from one place to another (environmental contamination), and applying effective sanitary procedures such as pasteurization, decontamination, sanitization, disinfection, and sterilization.

**Table 1:** Characteristics of the research group.

Total responders number n=185								
		Place of work						
		Residential care and treatment center No.1		Residential care and treatment center No.2		Residential care and treatment center No.3		p-value
		n	%	n	%	n	%	
Sex	Female	15	51,72	47	92,16	95	91,35	<0,001
	Male	14	48,28	4	7,84	9	8,65	
Education	Primary	0	-	3	6,00%	2	1,96%	0,758
	Vocational	0	-	8	16,00%	10	9,80%	
	Secondary	0	-	31	62,00%	68	66,67%	
	Higher	0	-	8	16,00%	22	21,57%	

2)Assessment of staff’s knowledge on preventing SARS-CoV-2 infection.

The own-authored questionnaire, developed jointly with all the study co-authors, based on the guidelines, information available on the WHO website, Polish government web sides, CDC (Centers for Disease Control and Prevention) and consisted of 34 questions. The first 16 questions concerned the verification of knowledge about the methods of transmission and prevention of SARS-CoV-2 virus infections. In this part, verification of knowledge was based on confirmation or denial of certain statements In this part there were 3 answers possible to choose: I agree, I disagree, I do not know of which only one was correct. On the other hand, the next 18 questions concerned general knowledge related to the SARS-CoV-2 virus. This part of the questionnaire contained 4 answers to choose from, of which only one was correct. Participant could earn 1 point for each correct answer. The maximum number of points was 34 (if 34 answers were correct). In accordance with survey design principles, comprehension of all questions was checked on a group of 20 individuals (pre-study) after the survey was created. None of the respondents made any comments regarding the design of the questions or problems with understanding. The next step was to use the created survey questionnaire to survey the target population- medical workers. The survey showed good psychometric properties. Cronbach’s alpha coefficient was greater than 0.8, which means that the survey is a good research tool.

3)Implementation of procedures to limit the spread of SARS-CoV-2

4)Series of training sessions targeting the identified knowledge deficits

The analysis of survey results was followed by the selection, in cooperation with the management, of priority areas in which education should be provided to the group studied. Mandatory training was planned over the next two months, focusing on the main topics found to be problematic based on the surveys.

5)Evaluation of the implemented procedures and their effectiveness in day-to-day operations.

## RESULTS

The project was attended by 185 medical workers (nurses, medical carers, physiotherapists and doctors). The majority of the survey participants were female and had a secondary education. The average age of the respondents was 45,7 (SD=9,5) (Table 1). The study had 4 steps. All the steps taken covered the period from May 2020 to October 2020, which corresponds to the period of the first wave of covid-19 disease in Poland.

### Step 1. Audit of existing hygiene procedures used to limit the risk of SARS-CoV-2 spread

Our audit of the applied procedures determined that many of them, despite a properly defined objective, were poorly planned and implemented. The most commonly detected shortcomings included:

(1) incorrectly selected disinfectants for skin and equipment (incorrect spectrum of activity, too long contact time, too high concentrations causing risk to staff and residents);
(2) incorrectly selected cleaning and decontamination products for surfaces and rooms, with a spectrum not including coronaviruses;
(3) lack of procedures for medical waste;
(4) lack of procedures for isolation in case of suspected or confirmed infection;
(5) lack of procedures regulating the use of personal protective equipment when working with a suspected SARS-CoV-2 patient;
(6) lack of procedures for managing patients with suspected infection or admitted without an RT-PCR swab and after contact with an infected person.

The results of our audit were discussed in a meeting with the management, which also included an assessment of the current epidemiological situation, including any problems that may disrupt the safe operation of the facilities during the SARS-CoV-2 pandemic. Significant problems were unanimously identified regarding knowledge on safe working practices and procedures. There were also concerns among the staff about the infection risk, while the recommended PPE was not correctly used. Follow-up actions were introduced, aiming at improving and completing the procedures that form a solid foundation for safe work during the SARS-CoV-2 pandemic.

**Table 2:** Total score in the knowledge test administered to staff in facilities providing care for senior citizens and people with disabilities.

Group n=185								
	Place of work						P-value	P-value (multiple comparisons)**
	Residential care and treatment center No.1		Residential care and treatment center No.2		Residential care and treatment center No.3			
	x¯	SD	x¯	SD	x¯	SD		
Test result	8.89	2.5	10.48	3.3	10.08	2.4	0.039	Place 1 vs Place 2: p=0.030 Place 1 vs Place 3: p=0.090 Place 2 vs Place 3: p=0.649

x¯ — mean; SD — standard deviation; ** Tukey’s test

### Step 2. Analysis of knowledge among the staff in facilities providing care for senior citizens and people with disabilities

The knowledge of staff in facilities providing care for senior citizens and people with disabilities was assessed using surveys. This assessment demonstrated that on average, the knowledge level was low, with scores ranging between 8.89 and 10.8 points. Statistically significant differences were found between the staff from center No. 1 vs staff from center No. 2 (p=0.039) (Table 2). It was observed that employees from all 3 medical facilities showed a lack of knowledge on how to deal with the occurrence of covid-19 symptoms in patients, how to deal with the patient in the case of a positive SARS-CoV-2 result and how to deal with the symptoms of infection in people with medical personnel. The knowledge connected with methods of SARS-CoV-2 virus transmission was also poor.

Most correct answers were provided to questions regarding personal hygiene and socially responsible behavior, similarly to those on virus spread, hand washing, avoidance of physical contact and touching one’s face and nose and covering one’s mouth during sneezing and coughing. 90% of respondents in all the facilities studied answered these questions correctly. More difficulties were associated with questions regarding SARS-CoV-2 infection risk groups and those most likely to develop a severe form of COVID-19. The staff of the analyzed care facilities did not know that anyone, regardless of age and health, can become infected, or that elderly patients and those with comorbidities most often develop severe forms of COVID-19. Responders had a problem with defining the duration of hand disinfection. In this topic, the highest number of people from facility No. 2 demonstrated knowledge in this area (78%) compared to facility 1 and 3 where the number of correct answers were respectively: 54% and 35%. Almost half of the surveyed medical centers (center No. 1 (52%) vs. center No. 2 (44%) vs. center No. 3(59%)) believed that elderly people usually do not show symptoms of SARS-CoV-2 infection. In the facility No. 1, 70% of respondents did know that SARS-CoV-2 can be transmitted via the oral route compared to center No. 2 and No. 3 where knowledge about it showed 28,9% and 24,8% responders, respectively (Table 3).

The next part of the survey showed that the respondents knew the main symptoms of COVID-19, the incubation time of the virus, and the treatment options. Respondents knew the recommended disinfection time, but did not know the optimal concentration of disinfectant. Sadly, the staff did not correctly understand the most common terms for administrative restrictions, such as quarantine and isolation, or the currently recommended procedures regulated by medical policy. The staff found it difficult to define terms such as low-risk and high-risk exposure, or procedures to follow in case of professional exposure or patient exposure. Also, in facility No. 1, the least among the studied group (67%) knew that people with comorbidities were at risk of covid-19 compared to respondents from facility No. 2 and 3, where, respectively, 100% and 93% had such knowledge. Only 33.3% of the staff of facility No. 1 knew how to deal with a patient who will present symptoms such as temp >38 degrees with coughing or dyspnea while in institution No. 2 and 3, this knowledge was slightly higher at 44% and 60% respectively (Table 4).

**Table 3:** Knowledge on SARS-CoV-2 prevention and transmission.

Number of responders n=185								
		Place of work						P -value
		Residential care and treatment center No.1		Residential care and treatment center No. 2		Residential care and treatment center No. 3		
		n	%	n	%	n	%	
1. Maintaining personal hygiene and behaving in a socially responsible manner prevents the spread of SARS-CoV-2 Correct answer: I agree	I agree	29	100,00	50	98,04	102	97,15	0,692
	I disagree	0	0,00	1	1,96	1	0,95	
	I do not know	0	0,00	0	0,00	2	1,90	
2. Coronavirus is transmitted between humans, mainly through respiratory droplets Correct answer: I agree	I agree	28	96,55	49	96,08	101	96,19	0,413
	I disagree	1	3,45	2	3,92	1	0,95	
	I do not know	0	0,00	0	0,00	3	2,86	
3. Coronavirus can also be transmitted through the alimentary route Correct answer: I agree	I agree	19	70,37	13	28,89	26	24,76	<0,001
	I disagree	2	7,41	27	60,00	64	60,95	
	I do not know	6	22,22	5	11,11	15	14,29	
4. Frequent hand washing with soap or disinfectant prevents the spread of SARS-CoV-2 Correct answer: I agree	I agree	27	93,10	51	100,00	103	98,10	0,139
	I disagree	2	6,90	0	0,00	1	0,95	
	I do not know	0	0,00	0	0,00	1	0,95	
5. Avoidance of physical contact when greeting someone prevents the spread of SARS-CoV-2 Correct answer: I agree	I agree	28	96,55	50	98,04	102	97,15	0,704
	I disagree	0	0,00	1	1,96	1	0,95	
	I do not know	1	3,45	0	0,00	2	1,90	
6. Avoidance of touching one’s eyes, nose, and mouth with one’s hands prevents the spread of SARS-CoV-2 Correct answer: I agree	I agree	29	100,00	51	100,00	103	98,10	0,819
	I disagree	0	0,00	0	0,00	1	0,95	
	I do not know	0	0,00	0	0,00	1	0,95	
7. Covering one’s mouth with one’s elbow or clothing when coughing or sneezing is a good practice for preventing the spread of SARS-CoV-2 Correct answer: I agree	I agree	29	100,00	43	84,31	101	96,20	0,003
	I disagree	0	0,00	8	15,69	2	1,90	
	I do not know	0	0,00	0	0,00	2	1,90	
8. Wearing a face mask during contact with infected individuals protects against SARS-CoV-2 infection Correct answer: I agree	I agree	26	92,86	46	90,20	100	95,24	0,365
	I disagree	2	7,14	4	7,84	2	1,90	
	I do not know	0	0,00	1	1,96	3	2,86	
9. Following social distancing rules and avoiding crowded spaces limits the spread of SARS-CoV-2 Correct answer: I agree	I agree	28	96,55	50	98,04	104	99,05	0,626
	I disagree	1	3,45	1	1,96	1	0,95	
	I do not know	0	0,00	0	0,00	0	0,00	
10. Wearing a face mask is considered an appropriate protective measure against SARS-CoV-2 for a person without COVID-19 symptoms Correct answer: I disagree	I agree	28	96,55	51	100,00	101	96,19	0,353
	I disagree	1	3,45	0	0,00	1	0,95	
	I do not know	0	0,00	0	0,00	3	2,86	
11. Correct use of a face mask during an epidemic should include covering one’s nose, mouth, and chin, with the colored side of the mask facing outwards Correct answer: I agree	I agree	27	93,10	43	86,00	103	98,10	0,006
	I disagree	1	3,45	7	14,00	1	0,95	
	I do not know	1	3,45	0	0,00	1	0,95	
12. Staying at home plays an important role in preventing the spread of SARS-CoV-2 Correct answer: I agree	I agree	26	89,66	50	100,00	97	92,39	0,189
	I disagree	3	10,34	0	0,00	6	5,71	
	I do not know	0	0,00	0	0,00	2	1,90	
13. Individuals with pre-existing chronic diseases (heart disease, diabetes, high blood pressure, cancers, autoimmune diseases) are at a higher risk of contracting SARS-CoV-2 Correct answer: I agree	I agree	20	68,97	51	100,00	98	93,34	<0,001
	I disagree	9	31,03	0	0,00	5	4,76	
	I do not know	0	0,00	0	0,00	2	1,90	
14. SARS-CoV-2 is mainly contracted by elderly individuals Correct answer: I disagree	I agree	8	27,58	9	17,65	18	17,14	0,496
	I disagree	20	68,97	42	82,35	84	80,00	
	I do not know	1	3,45	0	0,00	3	2,86	
15. Elderly individuals infected with SARS-CoV-2 typically have no symptoms Correct answer: I disagree	I agree	15	51,72	22	44,00	62	59,05	0,460
	I disagree	12	41,38	25	50,00	36	34,28	
	I do not know	2	6,90	3	6,00	7	6,67	
16. Young people do not contract SARS-CoV-2 Correct answer: I agree	I agree	2	7,14	1	1,96	8	7,62	0,350
	I disagree	26	92,86	50	98,04	94	89,52	
	I do not know	0	0,00	0	0,00	3	2,86	

### Step 3. Training stage

Training sessions were successfully provided to 90% of the nurses, carers, rehabilitation specialists, and physicians. A significant amount of time was also devoted to educational meetings for managers. Attitudes observed among the staff during training included concern about the infection and reluctance towards change. In the training, participants were encouraged to ask questions, and to discuss, within each team, the changes to procedures, possibilities of implementation and any resulting problems, and the everyday difficulties arising during the pandemic. For many participants, the procedures to apply after exposure and a positive RT-PCR test result were unclear. In each facility, the training also focused on the clinical picture of the infection, and staff were made aware of the need to correctly identify the varying symptoms of the disease, including ones that are less common and could be mistaken for non-COVID symptoms, resulting in the “release” of infection among the patients residing in the building.

### Step 4. Updates and implementation of procedures

Our audit of existing procedures demonstrated that, despite the staff’s commitment, some procedures required modification. Additional procedures were also introduced to enable safe work in teams rotating every week, so as to prevent contact between the teams. This solution would enable continuation of care for patients even in the event of infections among the staff.

Procedures introduced:

(1)Three zones have been created (procedure for managing suspected or confirmed risk of SARS-CoV-2 infection): green for infection-free individuals, orange for those who have had high-risk exposure, and red for those with symptoms. The rules for the zone system were precisely defined. Patients remain in the red zone until their RT-PCR results come back. In case of a positive test, arrangements should be immediately made to transfer the patient to an infectious disease hospital or another designated place, based on the so-called “social indications”, *i.e*., to prevent other residents from contracting the infection. Whenever a patient who had required hospitalization returns to the facility or a new patient is admitted, the standard procedure involves performing an RT-PCR test and isolating the patient for 7 days to rule out any recent infection. Separate staff teams work in each zone to prevent cross-contact and contamination. The rules for the use of personal protective equipment, providing patient care, and for disposing of uneaten food were strictly regulated.

(2)Another procedure implemented concerned cases of epidemiological hazard due to the coronavirus among the staff. This procedure defined high- and low-risk exposure rules and the course of action in applicable cases. In each facility, one employee was designated to perform an epidemiological investigation in cases of staff or patient exposure, trace contacts in each of the risk groups, provide instructions for further actions, and notify the sanitary inspectors. A symptom monitoring chart has been added to the documentation. Employees were obliged to fill out the symptom monitoring chart for each patient twice a day, and if anything alarming happened, to respond immediately by placing the patient in red zone isolation, ordering an RT-PCR test, and identifying all at-risk individuals.

**Table 4:** Analysis of respondents’ knowledge.

Number of responders n=185								
		Place of work						p- value
		Residential care and treatment center No. 1		Residential care and treatment center No. 2		Residential care and treatment center No.3		
		n	%	n	%	n	%	
1. Which of the following are the three main symptoms of Covid-19? Correct answer: fever, dry cough, shortness of breath	Correct	18	64,29	48	96,00	87	82,86	0,001
	Incorrect	10	35,71	2	4,00	18	17,14	
2. What is the average incubation period of coronavirus? Correct answer: 2-14 days	Correct	18	64,29	26	54,17	54	51,92	0,506
	Incorrect	10	35,71	22	45,83	50	48,08	
3. What are the available medications and treatment options? Correct answer: Medication is not available	Correct	8	28,57	13	27,66	50	49,50	0,016
	Incorrect	20	71,43	34	72,34	51	50,50	
4. What is the correct disinfection time? Correct answer: 15-30 seconds	Correct	14	53,85	36	78,26	35	35,00	<0,001
	Incorrect	12	46,15	10	21,74	65	65,00	
5. What is the optimal concentration of alcohol for hand disinfection? Correct answer: Above 60%	Correct	22	78,57	44	88,00	78	78,79	0,363
	Incorrect	6	21,43	6	12,00	21	21,21	
6.What does „social distancing” mean in relations to the prevention of infectious disease transmission? Correct answer: Nonpharmaceutical actions or measures taken to prevent the spread of a communicable disease by maintaining a physical distance of at least 2 meters between people.	Correct	21	75,00	46	92,00	94	90,38	0,051
	Incorrect	7	25,00	4	8,00	10	9,62	
7. What does „quarantine” mean and who is subjected to it? Correct answer: seclusion of a healthy person who has been exposed to an infection to prevent the spread of particularly dangerous and highly contagious diseases	Correct	16	59,26	36	72,00	45	43,27	0,003
	Incorrect	11	40,74	14	28,00	59	56,73	
8. What measures should be used when one is exposed to or in continued contact with a source of SARS-COV-2 infection? Correct answer: Quarantine or epidemiological surveillance	Correct	21	77,78	19	38,00	43	41,75	0,002
	Incorrect	6	22,22	31	62,00	60	58,25	
9. Individuals with a suspected or confirmed SARS-CoV-2 infection or COVID-19 disease should be subjected to: Correct answer: be removed from duty until the patient’s test results are known	Correct	6	21,43	15	30,00	6	5,83	<0,001
	Incorrect	22	78,57	35	70,00	97	94,17	
10. What should be done once a patient with a suspected SARS-CoV-2 infection is transferred to an infectious disease hospital? Correct answer: All correct	Correct	22	81,48	33	66,00	47	45,63	0,001
	Incorrect	5	18,52	17	34,00	56	54,37	
11. Staff who had been in close contact with a patient without the proper protective equipment should: Correct answer: be removed from duty until the patient’s test results are known	Correct	3	11,11	3	6,00	6	5,83	0,603
	Incorrect	24	88,89	47	94,00	97	94,17	
12. Close contact is defined as: Correct answer: All correct	Correct	7	28,00	40	81,63	92	88,46	<0,001
	Incorrect	18	72,00	9	18,37	12	11,54	
13. Each patient showing symptoms of acute respiratory infection (fever over 38°C with coughing or dyspnea) in combination with epidemiological criteria should be admitted to an infectious disease department or infectious disease observation unit: Correct answer: Yes	Correct	9	33,33	22	44,00	62	59,62	0,024
	Incorrect	18	66,67	28	56,00	42	40,38	
14. How is SARS-CoV-2 transmitted? Correct answer: a and c (droplet and faecal-oral route)	Correct	14	51,85	33	66,00	72	69,23	0,237
	Incorrect	13	48,15	17	34,00	32	30,77	
15. Among commonly used objects, which carry the highest risk of SARS-CoV-2 transmission? Correct answer: Mobile phone	Correct	1	3,70	7	14,29	33	31,73	0,002
	Incorrect	26	96,30	42	85,71	71	68,27	
16. Contact with low-risk exposure is: Correct answer: contact with the infected at a distance >2m and a time < 15min. Longer contact increases the risk of transmission; the 15 minute time period is arbitrarily set. It may be that the degree of risk will be determined on a case- by-case basis and action will be taken even for shorter contacts	Correct	16	64,00	24	48,00	68	66,67	0,081
	Incorrect	9	36,00	26	52,00	34	33,33	
17. If an employee shows symptoms such as fever, coughing, shortness of breath during work, the following should be done. Correct answer: Immediate notification of supervisor and infection control team, withdrawal from work, identification of contact people, isolation until smear results are available, further action depending on results	Correct	17	62,96	41	82,00	91	87,50	0,012
	Incorrect	10	37,04	9	18,00	13	12,50	
18. In the event of infection, medical personnel under home isolation: Correct answer: cannot leave the house due to isolation, is waiting at home for the intervention team to arrive to take a swab, or is following the recommendations of the Sanitary Inspectoriate	Correct	16	59,26	38	76,00	95	91,35	<0,001
	Incorrect	11	40,74	12	24,00	9	8,65	

(3)An important part of the new measures involved defining “Ten guidelines for safe work in a nursing home”, which were recognized as a gold standard. These guidelines included wearing masks at all times in the facility, hand disinfection, and monitoring temperature and other symptoms that could require one to stay at home. During shifts, employees were required to combine tasks performed with each patient to limit contact time to 15 minutes. Staff were also made aware of other potential sources of infection, such as commuting together or meeting socially outside of work. All staff were required to learn and implement the new procedures.

(4)Additionally, a decontamination procedure for rooms, beds, and equipment was introduced, specifying the products to use and their proper concentration. The staff were trained in the new procedure and monitored throughout the implementation period and for the first three months of application. During the final meeting, which took place during the second wave of the pandemic, the new procedures were reviewed, and they were reported as very beneficial and effective as a COVID-19 control measure. At the beginning of the second wave of the pandemic, antigen tests were introduced for rapid outbreak identification and quick response. As the waiting period is longer in the case of RT-PCR tests, a positive antigen test result allowed for the person to be isolated until an RT-PCR test could be completed.

All the additional restrictions were accepted by the staff, who developed a strong sense of responsibility for the safety of patient care they provided.

## DISCUSSION

The SARS-CoV-2 pandemic continues, and knowledge on the virus and the mechanisms through which it causes severe illness is still extremely limited. Some early observations, as well as knowledge gained during the previous SARS-CoV-1 epidemic, suggest that monocytes and monocyte-derived alveolar macrophages come into play early and are key in the progression to severe COVID-19, contributing to a cytokine storm, ARDS, and diffuse peripheral tissue damage. The pathological response of monocytes in COVID-19 shows some similarities to that associated with aging, which suggests that monocytes can contribute to the disproportionately greater severity of COVID-19 in the elderly [13, 20]. Much further experimental research is needed to test this and other hypotheses thoroughly. Undoubtedly, until full knowledge is gained, all the available prevention and containment methods must be used. It is well said that “prevention is better than cure” [21].

The knowledge and awareness of medical personnel regarding the prevention of SARS-CoV-2 spread is crucial [22]. Sadly, our study has demonstrated knowledge deficits and limited capabilities of providing safe care to elderly patients. It seems that the identified shortcomings have contributed to infections and transmission of the virus among members of the staff and patients. The few available publications have often reported gaps in knowledge on SARS-CoV-2 both in medical personnel and medical students, and in the general society [22-24]. Certain socio-clinical variables are thought to be associated with knowledge level. Better knowledge was found in more educated, professionally active individuals, with higher monthly incomes [25]. In our study, few respondents had completed higher education, while the earnings in this sector of healthcare are ranked among the lowest, which may confirm the theory cited above.

As in our study, others have found that wearing a face mask is the most popular and recognized form of protection against SARS-CoV-2, similar to hand washing, social distancing, and using alcohol-based disinfectants. Unfortunately, medical staff in our study did not have the expected level of knowledge on virus transmission routes, administrative terms associated with infectious disease spread, or the risk groups for infection and full-blown COVID-19. The respondents in our survey were not aware that young people may be infected at the same rate as the elderly, though senior citizens are at more risk of becoming severely ill. The few studies published around the world report similar levels of knowledge in medical staff, with similar deficits, which tend to decrease as education level increases [26].

Research on a COVID-19 vaccine is underway around the world. The latest reports indicate that the first SARS-CoV-2 vaccine used in Europe will be the Pfizer vaccine, approved in the UK. The product has successfully passed through all clinical trial phases with satisfactory results, especially in the final phase of trials. Its effectiveness has been confirmed at a level of 95%, which gives hope for containing the SARS-CoV-2 pandemic, provided that a sufficiently large portion of the population is vaccinated, especially in the group most at risk of severe illness and death [27, 28]. In Poland, a strong anti-vaccination movement exists, giving rise to concerns about widespread unwillingness to get the SARS-CoV-2 vaccination even when it becomes available. According to the latest survey by the CBOS polling institute, carried out between November 5 and 15, 2020, “more than one in three respondents (36%) would get vaccinated against COVID-19, if a vaccine were available, while nearly half would be opposed to this (47%, including 27% strongly opposed). A large number of respondents were still undecided (17%).” The collected data demonstrate that attitudes towards the COVID-19 vaccination largely depend on the respondent’s age. Those aged 45 and above were more likely to get vaccinated than younger respondents, and the oldest ones, aged 65 and above, were the most in favor of vaccination (49%, including 26% strongly in favor) (www.cbos.pl/PL/publikacje/news/2020/41/newsletter.php, Accessed 5 Dec 2020). In the light of these results, the implementation of other, “non-invasive” methods of COVID-19 prevention, as well as procedures limiting SARS-CoV-2 infections and transmission, seems warranted. Our study demonstrated that increased knowledge among medical personnel translated into fewer infections, and the implemented procedures, educational efforts, and effectiveness monitoring processes improved the safety of staff working in the facilities studied and in the patients residing there.

For mid-level personnel, five areas of responsibility have been designated during the COVID-19 pandemic: (1) health education, prevention, and support; (2) prevention and detection of hospital-acquired infections; (3) planning and implementing personal protection measures; (4) protecting immunodeficient or severely ill individuals; (5) providing care to patients with COVID-19. This multitude of tasks requires nurses to have the relevant knowledge allowing them to perform their duties efficiently and correctly [30]. In our study group, an educational intervention included 90% of the staff working in the care facilities analyzed. For comparison, in the study by Semerci et al. (2020), education was only provided to one in two nurses [29], and the authors reported that this was not sufficient for controlling and preventing infections. Chen et al. stated that nurses must receive training on the prevention and management of COVID-19, including the proper use of personal protective equipment, detection of early symptoms and signs of infection, proper personal hygiene practices, and corresponding environmental measures [30]. Needless to say, appropriate education should be provided to everyone involved: staff, patients, and support services. The COVID-19 guidelines published by the Ministry of Health certainly contribute towards safe working practices. In Poland, no guidelines were available during the initial period, and most healthcare professionals followed the information published by the World Health Organization. As knowledge on COVID-19 remains rather limited, healthcare professionals should feel the need to regularly check all available guidelines, from the standards applied in their place of work to those published by the Ministry of Health [31].

Efforts to raise awareness and implement safe working practices are the foundation of our fight against the pandemic. The way that changes are implemented is extremely important, as each change must be fully understood, expected, and acceptable to those affected. Good practice standards create a sense of security both among the staff and the patients. According to Roy et al. [32], a good level of awareness often leads to optimistic attitudes, which may have a positive influence on the preparedness of medical personnel to solve problems related to the pandemic [32].

### Study limitations

The questionnaire for our study was developed based on the information available on WHO, CDC, and Polish government websites. The main purpose of the study was to demonstrate the needs related to the management of care for the elderly during a pandemic. The knowledge of staff in care facilities is just one of its components. Our respondents’ group was limited to those employed in the senior- and chronic care sector. The study was conducted in 3 public medical centers but in the same area. Another limitation is the lack of reassessment of respondents’ knowledge after the post-training activities introduced. However, the purpose of this study was to assess the knowledge of staff employed in elder care that was helpful to initiate actions and implement existing procedures. The knowledge assessment was the starting point for implementing any measures to reduce the risk of SARS-CoV-2 transmission. Due to the lengthy process of implementing the procedures and obtaining the effects of the implemented actions, a reassessment of knowledge was not conducted during the preparation of this manuscript.

## References

[1] Dare RK, Fry AM, Chittaganpitch M, Sawanpanyalert P, Olsen SJ, Erdman DD (2007). Human Coronavirus Infections in Rural Thailand: A Comprehensive Study Using Real-Time Reverse-Transcription Polymerase Chain Reaction Assays. J Infect Dis, 196:1321-8. 17922396 10.1086/521308 PMC7109921

[2] Ye ZW, Yuan S, Yuen KS, Fung SY, Chan CP, Jin DY (2020). Zoonotic origins of human coronaviruses. Int J Biol, 16.10:1686. 10.7150/ijbs.45472 PMC7098031 32226286

[3] Ashour HM, Elkhatib WF, Rahman M, Elshabrawy HA (2020). Insights into the recent 2019 novel coronavirus (SARS-CoV-2) in light of past human coronavirus outbreaks. Pathogens, 9.3:186. 10.3390/pathogens9030186 PMC7157630 32143502

[4] Xu J, Zhao S, Teng T, Abdalla AE, Zhu W, Xie L, et al (2020). Systematic comparison of two animal-to-human transmitted human coronaviruses: SARS-CoV-2 and SARS-CoV. Viruses, 12.2:244. 10.3390/v12020244 PMC7077191 32098422

[5] Banik GR, Alqahtani AS, Booy R, Rashid H (2016). Risk factors for severity and mortality in patients with MERS-CoV: Analysis of publicly available data from Saudi Arabia. Virol Sin, 31.1:81-84. 26826080 10.1007/s12250-015-3679-z PMC7090713

[6] Bansal M (2020). Cardiovascular disease and COVID-19. Diabetes Metab Syndr, 14.3:247-250. 32247212 10.1016/j.dsx.2020.03.013 PMC7102662

[7] Fagherazzi G, Goetzinger C, Rashid MA, Aguayo GA, Huiart L (2020). Digital health strategies to fight COVID-19 worldwide: challenges, recommendations, and a call for papers. J Med Internet Res, 22.6:e19284. 32501804 10.2196/19284 PMC7298971

[8] Phua J, Weng L, Ling L, Egi M, Lim CM, Divatia JV (2020). Intensive care management of coronavirus disease 2019 (COVID-19): challenges and recommendations. Lancet Respir Med, 8.5:506-517. 32272080 10.1016/S2213-2600(20)30161-2 PMC7198848

[9] Xiong Y, Peng L (2020). Focusing on health-care providers’ experiences in the COVID-19 crisis. Lancet Glob Health, 8.6:e740-e741. 32573442 10.1016/S2214-109X(20)30214-X PMC7190304

[10] Amgain K, Neupane S, Panthi L, Thapaliya P (2020). Myths versus Truths regarding the Novel Coronavirus Disease (COVID-2019) Outbreak. J Karnali Acad Heal Sci, 3.1.

[11] Romano CM, Chebabo A, Levi JE (2020). Past, present, and future of COVID-19: a review. Braz J Med Biol Res, 53.9. 10.1590/1414-431X202010475 PMC7405018 32725080

[12] Garcez FB, Aliberti MJ, Poco PC, Hiratsuka M, Takahashi SDF, Coelho VA et al (2020). Delirium and adverse outcomes in hospitalized patients with COVID-19. J Am Geriatr Soc, 68.11:2440-2446. 32835425 10.1111/jgs.16803 PMC7460960

[13] Azkur AK, Akdis M, Azkur D, Sokolowska M, van de Veen W, Brüggen MC et al (2020). Immune response to SARS-CoV-2 and mechanisms of immunopathological changes in COVID-19. Allergy, 75.7:1564-1581. 32396996 10.1111/all.14364 PMC7272948

[14] Rello J, Storti E, Belliato M, Serrano R (2020). Clinical phenotypes of SARS-CoV-2: Implications for clinicians and researchers. Eur Respir J, 55.5. 10.1183/13993003.01028-2020 PMC7236837 32341111

[15] Hu B, Guo H, Zhou P, Shi ZL (2020). Characteristics of SARS-CoV-2 and COVID-19. Nat Rev Microbiol, 1-14. 33024307 10.1038/s41579-020-00459-7 PMC7537588

[16] Wu Z, McGoogan JM (2020). Characteristics of and Important Lessons from the Coronavirus Disease 2019 (COVID-19) Outbreak in China: Summary of a Report of 72 314 Cases From the Chinese Center for Disease Control and Prevention. JAMA, 323.13:1239-1242. 32091533 10.1001/jama.2020.2648

[17] Chowdhury SD, Oommen AM (2020). Epidemiology of COVID-19. J Dig Endosc, 11.1:3. 10.1055/s-0040-1712187 PMC7364648 40477080

[18] Bencivenga L, Rengo G, Varricchi G (2020). Elderly at time of COronaVIrus disease 2019 (COVID-19): possible role of immunosenescence and malnutrition. GeroScience, 42.4:1089-1092. 32578073 10.1007/s11357-020-00218-9 PMC7308600

[19] Galbadage T, Peterson BM, Gunasekera RS (2020). Does COVID-19 Spread Through Droplets Alone? Front Public Health, 8:163. 32391310 10.3389/fpubh.2020.00163 PMC7193306

[20] Pence BD (2020). Severe COVID-19 and aging: are monocytes the key? GeroScience, 42.4:1051-1061. 32556942 10.1007/s11357-020-00213-0 PMC7299454

[21] Ubani K (2020) COVID-19, Culture and Public Health Conditions in Developing Countries: Prevention Is Better Than Cure. Can Social Sci, 16.4:14-19.

[22] Ahmed N, Shakoor M, Vohra F, Abduljabbar T, Mariam Q, Rehman MA (2020). Knowledge, Awareness and Practice of Health care Professionals amid SARS-CoV-2, Corona Virus Disease Outbreak. Pak J Med Sci, 36.COVID19-S4:S49. 10.12669/pjms.36.COVID19-S4.2704 PMC7306948 32582314

[23] Papagiannis D, Malli F, Raptis DG, Papathanasiou IV, Fradelos EC, Daniil Z, et al (2020). Assessment of knowledge, attitudes, and practices towards new coronavirus (SARS-CoV-2) of health care professionals in Greece before the outbreak period. Int J Environ Res Public Health, 17.14:4925. 10.3390/ijerph17144925 PMC7400230 32650614

[24] Haque T, Hossain KM, Bhuiyan MMR, Ananna SA, Chowdhury SH, Islam MR, et al (2020). Knowledge, attitude and practices (KAP) towards COVID-19 and assessment of risks of infection by SARS-CoV-2 among the Bangladeshi population: An online cross sectional survey. Int J Biol Sci, 16(10): 1745-1752.32226294

[25] Serwaa D, Lamptey E, Appiah AB, Senkyire EK, Ameyaw JK (2020). Knowledge, risk perception and preparedness towards coronavirus disease-2019 (COVID-19) outbreak among Ghanaians: a quick online cross-sectional survey. Pan Afr Med J, 35.44. 10.11604/pamj.supp.2020.35.2.22630 PMC7875746 33623569

[26] Kamineni SRT, Balu P, Sivagananam P, Chellapandian P, Chelladurai UM, Jayasheelan VP, et al (2020). Knowledge of COVID-19 among nursing and Allied health care professionals working in tertiary care hospital. Int J Pharm Sci Res, 11:103-109.

[27] Mahase E (2020). Covid-19: Pfizer vaccine efficacy was 52% after first dose and 95% after second dose, paper shows. BMJ, 371:m4826. 33310706 10.1136/bmj.m4826

[28] Mahase E (2020). Covid-19: UK approves Pfizer and BioNTech vaccine with rollout due to start next week. BMJ, 371:m4714. 33268330 10.1136/bmj.m4714

[29] Semerci R, Kudubes AA, Eşref FÇ (2020). Assessment of Turkish oncology nurses’ knowledge regarding COVID-19 during the current outbreak in Turkey. Support Care Cancer, 1-8. 10.1007/s00520-020-05700-w PMC7443175 32829464

[30] Chen X, Liu Y, Gong Y, Guo X, Zuo M, Li J et al (2020). Perioperative management of patients infected with the novel coronavirus: recommendation from the Joint Task Force of the Chinese Society of Anesthesiology and the Chinese Association of Anesthesiologists. Anesthesiology, 132.6:1307-1316. 32195699 10.1097/ALN.0000000000003301 PMC7155907

[31] Cook TM, El-Boghdadly K, McGuire B, McNarry AF, Patel A, Higgs A, (2020). Consensus guidelines for managing the airway in patients with COVID-19: Guidelines from the Difficult Airway Society, the Association of Anaesthetists the Intensive Care Society, the Faculty of Intensive Care Medicine and the Royal College of Anaesthetists. Anaesthesia, 75.6:785-799. 32221970 10.1111/anae.15054 PMC7383579

[32] Roy D, Tripathy S, Kar SK, Sharma N, Verma SK, Kaushal V (2020). Study of knowledge, attitude, anxiety & perceived mental healthcare. Asian J Psychiatr. 51: 102083 10.1016/j.ajp.2020.102083 PMC7139237 32283510

